# A Selective *β*−Catenin‐Metadherin/CEACAM1‐CCL3 Axis Mediates Metastatic Heterogeneity upon Tumor–Macrophage Interaction

**DOI:** 10.1002/advs.202103230

**Published:** 2022-04-11

**Authors:** Sally K. Y. To, Maggie K. S. Tang, Yin Tong, Jiangwen Zhang, Karen K. L. Chan, Philip P. C. Ip, Jue Shi, Alice S. T. Wong

**Affiliations:** ^1^ School of Biological Sciences The University of Hong Kong Pokfulam Road Hong Kong China; ^2^ Laboratory for Synthetic Chemistry and Chemical Biology Limited 17W, Hong Kong Science and Technology Parks, New Territories Hong Kong China; ^3^ Department of Pathology The University of Hong Kong Queen Mary Hospital Pokfulam Road Hong Kong; ^4^ Department of Obstetrics & Gynaecology The University of Hong Kong Queen Mary Hospital Pokfulam Road Hong Kong China; ^5^ Centre for Quantitative Systems Biology and Department of Physics Hong Kong Baptist University Hong Kong China

**Keywords:** *β*‐catenin, cytokinesis, ovarian cancer, polyploid, tumor‐associated macrophages

## Abstract

Tumor heterogeneity plays a key role in cancer relapse and metastasis, however, the distinct cellular behaviors and kinetics of interactions among different cancer cell subclones and the tumor microenvironment are poorly understood. By profiling an isogenic model that resembles spontaneous human ovarian cancer metastasis with an highly metastatic (HM) and non‐metastatic (NM) tumor cell pair, one finds an upregulation of Wnt/*β*‐catenin signaling uniquely in HM. Using humanized immunocompetent mice, one shows for the first time that activated *β*‐catenin acts nonautonomously to modulate the immune microenvironment by enhancing infiltrating tumor‐associated macrophages (TAM) at the metastatic site. Single‐cell time‐lapse microscopy further reveals that upon contact with macrophages, a significant subset of HM, but not NM, becomes polyploid, a phenotype pivotal for tumor aggressiveness and therapy resistance. Moreover, HM, but not NM, polarizes macrophages to a TAM phenotype. Mechanistically, *β*‐catenin upregulates cancer cell surface metadherin, which communicates through CEACAM1 expressed on macrophages to produce CCL3. Tumor xenografts in humanized mice and clinical patient samples both corroborate the relevance of enhanced metastasis, TAM activation, and polyploidy in vivo. The results thus suggest that targeting the *β*‐catenin‐metadherin/CEACAM1‐CCL3 positive feedback cascade holds great therapeutic potential to disrupt polyploidization of the cancer subclones that drive metastasis.

## Graphical abstract

Macrophages are closely associated with cancer metastasis. Using an isogenic humanized mouse model, this study demonstrates that metastatic ovarian cancer cells can enhance the tumor‐promoting phenotypes of macrophages in contact. It further reports a novel phenomenon that these macrophages could reciprocally induce polyploidy in a subset of the metastatic cancer cells to promote tumor aggressiveness.

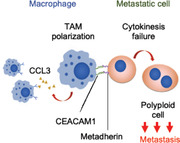

## 1 Introduction

Ovarian cancer is the fifth leading cause of cancer deaths among females.^[^ ^1^ ^]^ Most (70–75%) patients are diagnosed at an advanced stage with widespread peritoneal dissemination and malignant ascites, with a 5‐year survival rate of ≈30%. Peritoneal metastasis is notoriously difficult to treat due to its intimate and dynamic interactions with the tumor microenvironment.^[^ ^2^, ^3^ ^]^ The lack of suitable experimental models has bxeen one significant challenge to study the cellular and molecular mechanisms of this critical process, for example, the use of different human cancer cell lines has been complicated by their different genetic backgrounds and there is no non‐metastatic counterpart for eliminating bystander signaling events.

Emerging basic science and clinical findings suggest that interaction between tumors and their microenvironment, but not tumor cells, is key to tumor metastasis. However, cancers are heterogeneous not only genetically (interpatient heterogeneity), but also phenotypically (intratumor heterogeneity).^[^ ^4^, ^5^ ^]^ Although phenotypic heterogeneity and cell‐to‐cell variability are known to contribute to tumor relapse, they are often neglected in traditional bulk measurements due to technical complexity. Therefore, it is not clear whether different cancer cell subclones have different abilities to interact with and modulate the tumor microenvironment, especially the immune components.

Macrophages, the most abundant immune cells in the tumor microenvironment, play key roles in both the innate and adaptive immunity to orchestrate the concerted immune responses.^[^ ^6^ ^]^ Macrophages are functionally dynamic in response to microenvironmental cues. Two extreme macrophage polarization states are known as M1 and M2, which are analogous to type 1 and type 2 helper T‐cell responses—the former suppresses tumorigenesis and the latter promotes it.^[^ ^7^, ^8^ ^]^ Deciphering how macrophages can regulate metastasis is particularly critical in ovarian cancer for several reasons. First, disseminated cancer cells that succeed in infiltrating the peritoneum present distinct features of adhering to macrophages as immune aggregates in the omentum,^[^ ^9^ ^]^ which is the most common site of metastasis of ovarian cancer,^[^ ^3^ ^]^ whereas those fail to co‐opt also fail to thrive. Second, macrophages constitute ≈37% of all cell populations in the malignant ascites and a high density of TAMs significantly correlates with poor prognosis of ovarian cancer.^[^ ^10^, ^11^ ^]^ Third, depletion of macrophages, but not other immune cell types, resulted in reduced peritoneal metastasis and ascites formation in mice, hence further highlighting the important role of macrophages in ovarian cancer metastasis.^[^ ^12^ ^]^

In this study, we set out to investigate if metastatic cancer subclones could react distinctively to macrophage in contact and have a selective advantage for immunoediting, in addition to their intrinsically heightened migratory/invasive potential. Using the well‐controlled genotypically identical but phenotypically distinct highly metastatic (HM) and nonmetastatic (NM) cells in humanized mice, we show that *β*‐catenin, an important mediator of the oncogenic Wnt signaling, is a critical regulator in tumor–macrophage interactions at the metastatic site. Using single‐cell live imaging, we also provide evidence for a cell surface metadherin/CEACAM1‐CCL3 positive feedback loop which gives rise to the formation of polyploid tumor cells. The existence of these polyploid tumor cells in patient samples may underline the high level of aggressiveness and therapy resistance characteristic of metastasis.

## 2 Results

### 2.1 A Humanized Mouse Model Reveals a Role of *β*‐Catenin Signaling in Regulating Macrophages at the Metastatic Site

**Figure 1:**
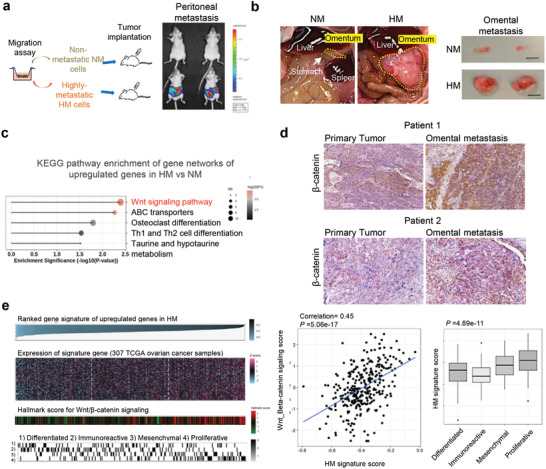
Wnt/*β*‐catenin signaling is enriched in the highly metastatic cells of the isogenic model and also in patient metastasis. a) The isogenic nonmetastatic NM and highly metastatic HM sublines were selected from the parental HEYA8 cell line using migration assays. The lines were intraperitoneally injected into nude mice and peritoneal metastasis was observed by bioluminescence imaging of luciferase activity. b) HM cells formed overt metastasis at the omentum of mice. Scale bar: 1 cm. c) Bioinformatic analysis on gene networks of upregulated genes (top 100 protein‐coding genes with the lowest FDR and the highest fold change) in HM versus NM shows an enrichment in the Wnt‐*β*‐catenin signaling pathway. d) Immunohistochemistry shows higher *β*‐catenin expression level in metastatic samples than in primary tumors from the same ovarian cancer patients. e) The HM gene signature score and the Wnt‐*β*‐catenin hallmark score were calculated for every sample in the TCGA ovarian cancer patient cohort by the ssGSEA method, and the correlation between the two scores was calculated (left and middle panel). The HM gene signature score was compared among the four ovarian cancer molecular subtypes (differentiated, immunoreactive, mesenchymal, and proliferative) (right panel).

To overcome current experimental limitations and iidentify key molecules in metastasis, we have successfully developed an isogenic ovarian cancer model of spontaneous metastasis, which closely resembles the metastatic events and molecular analyses found in human patients.^[^ ^13^ ^]^ Upon orthotopic injection at the ovarian bursa, HM possesses unique ability to metastasize consistently to the peritoneum, resembling human ovarian cancer dissemination. Conversely, NM was similarly tumorigenic, but it did not metastasize. Using intraperitoneal injection that mimics the metastatic route, we found that in addition to causing widespread peritoneal metastasis with tumor nodules forming at the intestinal mesenteries (**Figure** **1**a), HM cells also have a tendency for homing to the omentum (Figure 1b), which is consistent with clinical observations.^[^ ^3^ ^]^ We further analyzed NM and HM gene expression profiles using RNA sequencing. Bioinformatic analysis on the upregulated genes in HM versus NM has revealed significant enrichment in the Wnt‐*β*‐catenin signaling pathway in HM cells (Figure 1c). This is consistent with our previous findings using proteomics that showed activated *β*‐catenin signaling could enhance metastasis.^[^ ^13^ ^]^ While there are many studies on Wnt/*β*‐catenin signaling in the primary tumors of ovarian cancer patients, little is known about its role in the metastatic site of the same patients. We performed immunohistochemistry with 10 matched pairs of primary and metastatic high‐grade serous carcinoma tissues obtained from the same patients. As shown in Figure 1d, 60% showed stronger staining of *β*‐catenin in the metastatic site than in the primary site, implicating a role of activated

*β*‐catenin signaling during metastasis similarly in patients. We further calculated the HM gene signature score and the Wnt/*β*‐catenin hallmark gene set score for every sample in the TCGA ovarian cancer patient cohort using single‐sample Gene Set Enrichment Analysis (ssGSEA) and showed a positive correlation between the two scores in the ovarian cancer patient sample (TCGA dataset, 307 samples) (Figure 1e), highlighting the clinical relevance of our model. Gene expression profiling has identified four molecular subtypes of ovarian cancer, namely mesenchymal, immune, differentiated and proliferative, which differ in microenvironmental features and clinical outcomes.^[^ ^14^ ^]^ Interestingly, the HM signature score was found to be the lowest in the immunoreactive subtype, which is characterized by increased lymphocytes infiltration and is associated with the longest patient survival among the four subtypes (Figure 1e).

**Figure 2:**
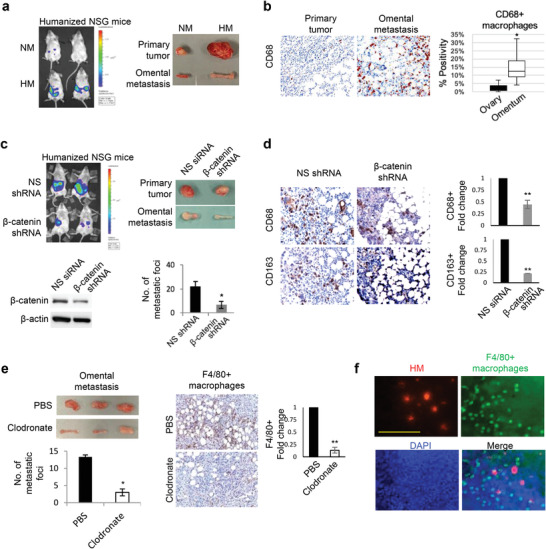
*β*‐catenin expression is associated with macrophages which promote ovarian cancer metastasis. a) NM and HM cells were orthotopically implanted into the ovarian bursa of humanized mice (3 mice per group). Peritoneal metastasis was monitored by bioluminescence imaging of luciferase activity. b) Immunohistochemistry was performed with the human macrophage marker CD68 on tumor sections from the primary site (ovary) and the omentum of mice orthotopically implanted with HM cells. The number of CD68+ macrophages was quantified. c) HM cells stably transfected with non‐specific (NS) or *β*‐catenin shRNA were orthotopically implanted into the ovarian bursa of humanized mice (3 mice per group). Peritoneal metastasis was monitored by bioluminescence imaging of luciferase activity. Omental metastasis and the number of mesenteric metastatic foci were also analyzed. Successful knockdown of *β*‐catenin was confirmed with Western blot. *β*‐actin serves as a loading control. d) Immunohistochemistry was performed on consecutive tumor sections to stain CD68+CD163+ tumor‐associated macrophages in the omentum, and their numbers were quantified and compared. e) Nude mice were i.p. injected with clodronate liposomes (twice a week for 2 weeks) to deplete macrophages. Control mice were treated with PBS liposomes (3 per group). Successful macrophage depletion by clodronate was confirmed by immunohistochemistry using mouse macrophage‐specific marker F4/80, followed by quantification with ImageJ. f) Immunofluorescence of fixed omentum shows close proximity between F4/80+ macrophages (green) and HM cells stainied with live‐cell tracker dye (red), which adhered to the omentum after intraperitoneal injection into nude mice for 4 hrs. Nuclei were counterstained with DAPI. *, *P* < 0.05. Scale bar: 100 µm.

The intrinsic role of *β*‐catenin signaling in promoting cancer growth and invasion has been widely studied; however, its extrinsic role in regulating the tumor microenvironment is still unclear, particularly in the interaction with immune cells. Despite deficiency in T cell development, nude mice have functional innate immunity consisting of macrophages, and have been widely used to study tumor–macrophage interactions^[^ ^15^, ^16^ ^.]^ However, the immune cells of human and mouse differ substantially in various aspects, including surface protein expression and cytokines/chemokines production.^[^ ^17^ ^]^ Therefore, to explore the interplay between *β*‐catenin and the immune microenvironment, we employed the humanized mouse model. In this model, CD34^+^ hematopoietic stem cells isolated from human cord blood were engrafted in irradiated immunodeficient NSG mice. The engraftment was considered successful with >25% hCD45^+^ cells in the peripheral blood. Our HM cell line retained its metastatic capability to colonize the omentum in this model (**Figure** **2**a), suggesting that HM harbors intrinsic factors that allow them to co‐opt with the functional human immune components. Given the importance of macrophages in ovarian cancers, we first compared the macrophage abundance in the primary tumor and the omentum metastasis and found a higher density of macrophages at the omentum metastases than at the primary site (Figure 2b). We next asked if the activated *β*‐catenin signaling could be an important factor for HM to survive in the macrophage‐rich environment. To this end, HM cells expressing non‐specific or *β*‐catenin shRNA were orthotopically injected into the ovarian bursa of these humanized mice. *β*‐catenin knockdown significantly decreased omentum metastasis and the number of metastatic nodules in these mice (≈3.2‐fold), and successful knockdown of *β*‐catenin was also confirmed by Western blot and immunohistochemical analysis (Figure 2c). Along with reduced metastasis, we observed reduced infiltration of CD68^+^CD163^+^ tumor‐associated macrophages in the omentum of mice injected with HM‐*β*‐catenin shRNA cells (Figure 2d). To pinpoint the role of macrophages in HM metastasis, we also used clodronate liposomes to deplete macrophages in nude mice.^[^ ^18^ ^]^ Intraperitoneal administration of clodronate liposomes led to a significant reduction of the omental metastasis and mesenteric metastatic nodules (≈fourfold) in mice as compared to the control PBS‐containing liposomes (Figure 2e). The depletion of macrophages by clodronate liposomes was confirmed by immunohistochemical staining of the murine macrophage marker F4/80, which was abundant in mice treated with control PBS liposomes (Figure 2e). These data confirm the specific and essential role of macrophages in mediating ovarian cancer metastasis in our isogenic model. We also intraperitoneally injected HM cells labeled with cell tracker dye and observed the close proximity of HM cells with omental macrophages upon their initial adhesion to the omentum (Figure 2f).

### 2.2 Live Cell Imaging Reveals the Induction of a Specific Polyploid Phenotype in HM Cells by Macrophages

**Figure 3:**
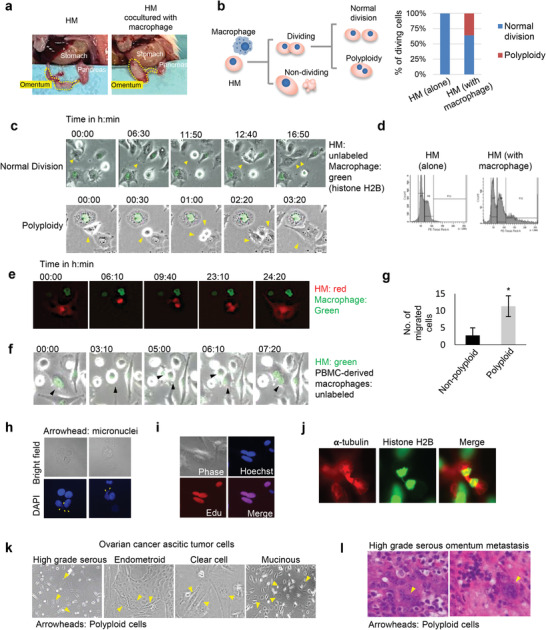
Macrophages induce polyploidy in metastatic cells. a) Coculture with THP1‐derived macrophages before tumor implantation enhanced HM metastasis in vivo. b) Different phenotypic changes were observed by time‐lapse imaging in HM cocultured with THP1 macrophages. At least 30 cells were traced for each group, and the percentages of HM cells undergoing normal division or polyploidization were shown. c) Representative time‐lapse images of different phenotypes of HM cocultured with macrophages were shown. Arrowheads indicate the same tumor cell throughout the imaging. d) DNA contents of HM cultured alone or isolated from macrophages coculture were analyzed with propidium iodide staining followed by flow cytometry. e) HM cells transfected with mCherry‐tubulin (red) were cocultured with macrophage stably expressing histone H2B‐GFP (green). f) Representative time‐lapse images of HM undergoing polyploidization during coculture with human PBMC‐derived macrophages were shown. g) Polyploid and nonpolylploid HM cells were flow sorted and subjected to migration assays. Migrated cells at the bottom of the chamber were fixed and stained with crystal violet. h) DAPI stain shows the presence of micronuclei in HM cocultured with THP1 macrophages. i) EDU staining (red) revealed that the co‐cultured HM could enter S phase of the cell cycle. j) Chromosome bridge was observed in a dividing HM (histone H2B‐GFP) upon the coculture. k) Bi‐ or multinucleated polyploid cells are present in ascitic cells from advanced‐stage ovarian cancers with different clinical subtypes. l) Representative H&E staining images shows polyploid cells present in the omental metastatic sample from an ovarian cancer patient. *, *P* <0.05.

To dissect the molecular basis of this HM‐driven tumor‐macrophage interaction in metastatic success and given the close spatial relation of these two cell types observed in vivo, we adopted a coculture assay in vitro to examine their interaction in detail. HM cells were either cultured alone or cocultured with THP1 cell‐derived macrophages.^[^ ^19^ ^]^ Isolated HM cells were then injected into nude mice intraperitoneally and omental metastasis was found to be more pronounced for HM with coculture, further confirming the metastasis‐promoting function of macrophages on HM cells (**Figure** **3**a). We further performed quantitative live single‐cell imaging to investigate the intercellular dynamics between cancer cells and macrophages. Macrophages were stably transduced with histone H2B‐GFP for the ease of tracking, while NM and HM remain unlabeled. No phototoxic effect was observed in THP1 alone, which is included in all the experiments as a control (Figure S1a, Supporting Information). By tracking individual cancer cells, we identified distinct growth phenotypes of NM and HM (Figure 3b). Whereas most (≈84%) NM cells are senescent or apoptotic (Figure S1b,c, Supporting Information), there are fewer HM cells (≈3%) that are apoptotic during the coculture, suggesting that HM has a better survival advantage than NM in the presence of macrophages. Notably, among all dividing cells, we observed a phenotype unique in HM, but not NM cells—a significant fraction (28%) of cells failed to complete cytokinesis and became polyploid (Figure 3b). These cells have undergone mitotic rounding, followed by formation and contraction of the cleavage furrow; however, the furrow finally regressed and they failed to split up into two daughter cells and fused to form an enlarged cell (Figure 3c; Video S1, Supporting Information). The increased DNA content in HM cells was validated with flow cytometry (Figure 3d). We also cocultured HM cells overexpressing *α*‐tubulin with THP1‐derived macrophages. The overexpressed tubulin remained within the HM cell failing cytokinesis throughout the imaging, suggesting that there is no transfer of cytoplasmic materials to its neighboring macrophages (Figure 3e). Together with the absence of GFP‐histone H2B in the polyploid HM cells, we confirmed that the phenotype is not due to a previously described mechanism of tumor–macrophage fusion.^[^ ^20^ ^]^ Polyploidy of HM cells was also observed upon coculture with macrophages derived from human peripheral blood mononuclear cells (Figure 3f). We also induced M2 macrophage differentiation with IL4 and performed the coculture experiment. Interestingly, time‐lapse imaging showed that M2 macrophages could support the formation of polyploid HM cells to a greater extent than M1 macrophages (Figure S2, Supporting Information), supporting the involvement of tumor‐associated M2 macrophages in regulating cancer polyploidy. We further isolated the polyploid cells by flow sorting followed by migration assays. We found that polyploid cells were more capable to migrate than those which are nonpolyploid (Figure 3g). In addition, we chemically induced polyploidy in HM using a polo‐kinase 4 inhibitor.^[^ ^21^ ^]^ HM cells treated with the inhibitor also exhibited greater migratory and tumor spheroid forming abilities (Figure S3a,b, Supporting Information). These data together suggest that polyploid HM cells are functionally more aggressive. Nuclear DAPI staining further revealed that the polyploid HM are bi‐ or multinucleated, with the presence of micronuclei (Figure 3h). Since a polyploid phenotype could either inhibit cancer cell growth by causing cell cycle arrest or promote tumor progression by enhancing genomic instability,^[^ ^22^ ^]^ we next investigated the proliferative potential of the polyploid HM cells. To exclude the possibility that these cells are in a senescent state, the senescence‐associated *β*‐galactosidase (SA‐*β*‐gal) staining was performed. The polyploid HM cells showed no or little staining, suggesting that they were non‐senescent (Figure S1c, Supporting Information). In contrast, NM cells cocultured with macrophages showed strong staining for SA‐*β*‐gal. EdU (5‐ethynyl‐2´‐deoxyuridine) proliferation assay was also carried out. EdU is a nucleoside analog of thymidine, which could be incorporated into DNA during DNA synthesis. Importantly, polyploid HM cells were positive for EdU staining, suggesting that they could enter the DNA synthesis phase of the cell cycle (Figure 3i). Furthermore, we also observed the formation of chromosome bridge in HM cells (Figure 3j), suggesting that furrow regression is induced by chromatin trapping. Intriguingly, polyploid cancer cells were found in tumor cells collected from fresh ovarian cancer patients’ ascites and also in FFPE samples of patients’ metastatic tumors (Figure 3k,l).

### 2.3 Macrophages Increase the Ploidy Level of HM through a Contact‐Dependent *β*‐Catenin/Metadherin Signaling

**Figure 4:**
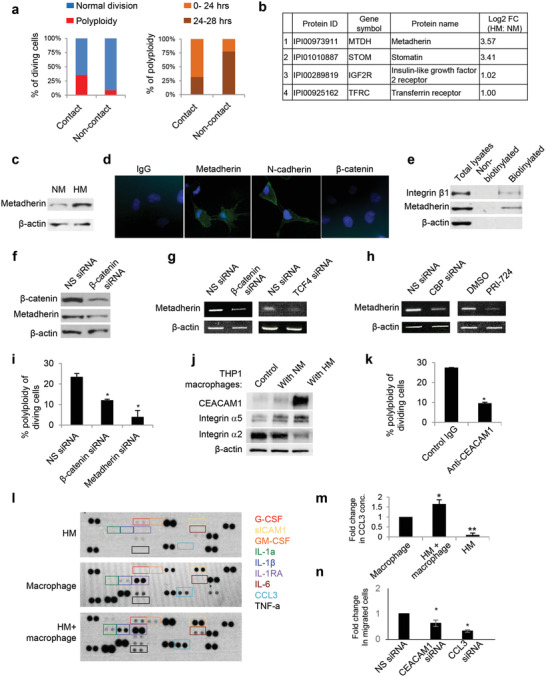
*β*‐catenin/metadherin in HM and CEACAM1 in macrophages promotes the polyploid phenotype. a) Polyploidy of HM occurred at higher frequency and earlier time in direct coculture of cells (contact) than in two‐chamber coculture (noncontact). b) Differentially expressed surface proteins in HM versus NM was detected by mass spectrometry. c) Western blot showed higher metadherin expression in HM compared to NM. d,e) The presence of membrane metadherin was confirmed by immunofluorescence and cell‐surface biotinylation followed by Western blot analysis. f) *β*‐catenin siRNA reduced metadherin protein expression in HM. g) HM cells were transfected with non‐specific (NS), *β*‐catenin or TCF4 siRNA. RT‐PCR was used to analyze the mRNA levels of metadherin. h) HM cells were transfected with NS or CBP siRNA, or treated with DMSO (vehicle control) or PRI‐724 (5 × 10^‐6^ m), a *β*‐catenin/CBP antagonist, for 48 h. RT‐PCR was used to analyze the mRNA levels of metadherin. i) Macrophages were cocultured with HM cells transfected with NS, *β*‐catenin, or metadherin siRNAs, and their interactions were captured with time‐lapse microscopy. Percentages of induced polyploidy in HM cells were shown. j) THP1 macrophages were cocultured with NM or HM cells. Expression levels of the indicated surface proteins in the isolated macrophages were analyzed by Western blot. k) THP1 macrophages were pretreated with CEACAM1 antibody, followed by HM coculture and time‐lapse imaging. Percentages of induced polyploidy in HM cells were shown. l) Conditioned media from HM, macrophage or coculture were analyzed with cytokine array. m) CCL3 levels in conditioned media from the indicated cultures were measured with ELISA assays. n) THP1 macrophages were transfected with CEACAM1 or CCL3 siRNA before the coculture. Cell culture supernatants were collected and used to induce the migration of THP1. *, *P* < 0.05, **, *P* < 0.01.

To evaluate if direct cell–cell contact is essential for the HM‐macrophage crosstalk, we employed a two‐chamber culture so that macrophages and HM cells were cultured with a shared tissue culture medium but without direct physical contact. There was a significant decrease in the percentage of the polyploid phenotype observed as compared to that with direct contact (**Figure** **4**a). This decrease was observed in terms of both the total number and the time required for polyploid induction, further suggesting the involvement of a contact‐dependent mechanism (Figure 4a).

To investigate novel contact‐dependent mechanism in HM–macrophage crosstalk, we specifically selected differentially expressed cell surface proteins with reported oncogenic functions between HM and NM from our previously reported mass spectrometry data.^[^ ^13^ ^]^ Among them, metadherin (IPI00973911) was found to have the highest fold change in expression in HM compared to NM cells and also in ascitic HM cells compared to primary HM cells (≈11‐ and 3‐fold increase respectively) (Figure 4b). The upregulation of metadherin in HM cells was confirmed by Western blot analysis (Figure 4c). While metadherin was reported to be a surface protein mediating lung metastasis of breast cancer,^[^ ^23^ ^]^ this protein has usually been reported to localize in other cellular compartments such as nucleus and endoplasmic reticulum.^[^ ^24^ ^]^ Using an antibody targeting an extracellular region of metadherin, our results indicated the cell surface localization of metadherin (Figure 4d). Immunostaining of the extracellular domain of N‐cadherin was used as a positive control, whereas intracellular *β*‐catenin was used as a negative control (Figure 4d). Cell surface protein biotinylation further confirmed the cell surface membrane localization of metadherin (Figure 4e). Given the activated *β*‐catenin signaling in HM cells, we asked if *β*‐catenin could regulate metadherin. Knocking down *β*‐catenin by specific siRNA in HM cells caused a marked decrease of metadherin protein expression (Figure 4f). Under active Wnt signaling, *β*‐catenin interacts with T‐cell factor 4 (TCF4) and many other cofactors to mediate transcriptional activation of various oncogenes.^[^ ^25^ ^]^ Therefore, we ask if *β*‐catenin may transcriptionally regulate metadherin. There was a clear reduction of metadherin mRNA by *β*‐catenin siRNA, confirming a specific role for *β*‐catenin (Figure 4g). Knockdown of TCF4 also significantly reduced metadherin mRNA levels (Figure 4g). CBP siRNA silencing and PRI‐724 antagonist treatment further suggest that the *β*‐catenin/TCF4 coactivator CREB‐binding protein (CBP)^[^ ^26^ ^]^ is involved (Figure 4h).

To decipher the role of *β*‐catenin/metadherin signaling in the interplay of HM cells and macrophages, we added HM cells transfected with nonspecific, *β*‐catenin, or metadherin siRNAs to the macrophage culture. Time‐lapse microscopy revealed a significant decrease in the polyploid phenotype among HM cells with either *β*‐catenin or metadherin silencing as compared to the non‐specific siRNA control (Figure 4i). Transfection of *β*‐catenin or metadherin siRNA did not cause failed cytokinesis in HM monoculture (data not shown).

### 2.4 CEACAM1 on Macrophages Is Important for the Interaction

The binding partner of surface metadherin remains largely unknown. We therefore analyzed the expression of several cell surface adhesion proteins and found an upregulation of CEACAM1 in macrophages induced by the presence of HM (Figure 4j). CEACAM1 is a type I membrane protein receptor variably expressed on the surface of different myeloid cells and lymphocytes.^[^ ^27^ ^]^ Considering the emerging regulatory roles of CEACAM1 in immune cells, we propose that the heterotypic interaction of CEACAM1/metadherin may contribute to the contact‐dependent communication between HM cells and macrophages. To test this, macrophages were pretreated with antibody that recognizes the extracellular domain of CEACAM1, which significantly reduced the polyploid phenotype (18%) as compared to IgG control during coculture (Figure 4k). CEACAM1 expression has been reported to be regulated by NF‐*κ*B signaling.^[^ ^28^ ^]^ Therefore, we transfected macrophages with siRNA against p65, a subunit of NF‐*κ*B. Interestingly, p65 siRNA significantly reduced the levels of CEACAM1, suggesting a role of NF‐*κ*B in CEACAM1 expression (Figure S4a, Supporting Information). We also treated HM cells with antibodies targeting the extracellular region of metadherin. There was a significant reduction of CEACAM1, suggesting that CEACAM1 increase is dependent on metadherin engagement (Figure S4b, Supporting Information). To test whether CEACAM1 alone promotes polyploidy, we used recombinant human (rh)CEACAM1 protein. We found that rhCEACAM1 was unable to promote polyploidy (Figure S4c, Supporting Information). Since CEACAM1 has various spliced isoforms and is a heavily glycosylated protein, we could not rule out the possibility that rhCEACAM1 differs from CEACAM1 expressed by macrophages and did not interact properly with metadherin. While the polyploidy formation might be dependent on CEACAM1, additional factors may also be needed for the phenomenon.

### 2.5 HM Induces TAM‐Like Phenotypes in Macrophages and Enhances CCL3 Level

We isolated the macrophages after 48 h of coculture and found that the presence of HM, but not NM, significantly increased transcript levels of CCL18, CCL22, IL‐1*β*, and IL‐6, which are cytokines well‐known to be enriched in TAMs,^[^ ^29^ ^]^ as compared to macrophages cultured alone (Figure S4d, Supporting Information). These suggest that HM, but not NM, has a selective advantage in skewing macrophages to a TAM‐like phenotype which may accelerate tumor progression. Moreover, we further performed a cytokine array analysis, and HM‐macrophages coculture caused a significant increase in many inflammatory cytokines (G‐CSF, GM‐CSF, sICAM1, IL‐*α*, IL‐1*β*, IL‐1RA, IL‐6, CCL3, and TNF*α*), which are known to be abundantly present in ovarian cancer ascites (Figure 4l).^[^ ^30^ ^]^

Chemokine (C–C motif) ligand 3 (CCL3) (also known as macrophage inflammatory protein 1 *α* (MIP‐1*α*)), is associated with metastasis of various tumor types such as renal cell and colorectal cancer,^[^ ^31^, ^32^, ^33^ ^]^ and was shown to mediate macrophage retention in the metastatic sites of breast cancer.^[^ ^35^ ^]^ Since the cytokine array analysis revealed a specific increase of CCL3 in the HM‐macrophage coculture, we next investigated the role of CCL3 in the crosstalk. We confirmed the upregulation of CCL3 by ELISA in the coculture (Figure 4m). CCL3 produced by HM cells alone is negligible, suggesting the macrophages are the major source of CCL3 production (Figure 4m). We also pretreated HM cells with antibodies targeting the extracellular region of metadherin before coculture. ELISA assays indicated a reduction in CCL3 upregulation, suggesting metadherin/CEACAM1 interaction is indeed needed for CCL3 induction (Figure S4e, Supporting Information). Time‐lapse imaging showed a significant reduction of polyploidy when the cocultured macrophages were transfected with CCL3 siRNA (Figure S4f, Supporting Information). When THP1 macrophages were allowed to migrate toward the conditioned medium (CM) collected from HM cells cocultured with macrophages, we found the coculture CM significantly enhances macrophage mobilization, and this effect was abolished when macrophages were transfected with CEACAM1 or CCL3 siRNA as compared to NS siRNA before the coculture (Figure 4n). These data suggest that CCL3 could play a role in attracting macrophages toward the interaction interface.

### 2.6 *β*‐Catenin/Metadherin and CEACAM1/CCL3 Signaling Are Important for Tumor Metastasis In Vivo

**Figure 5:**
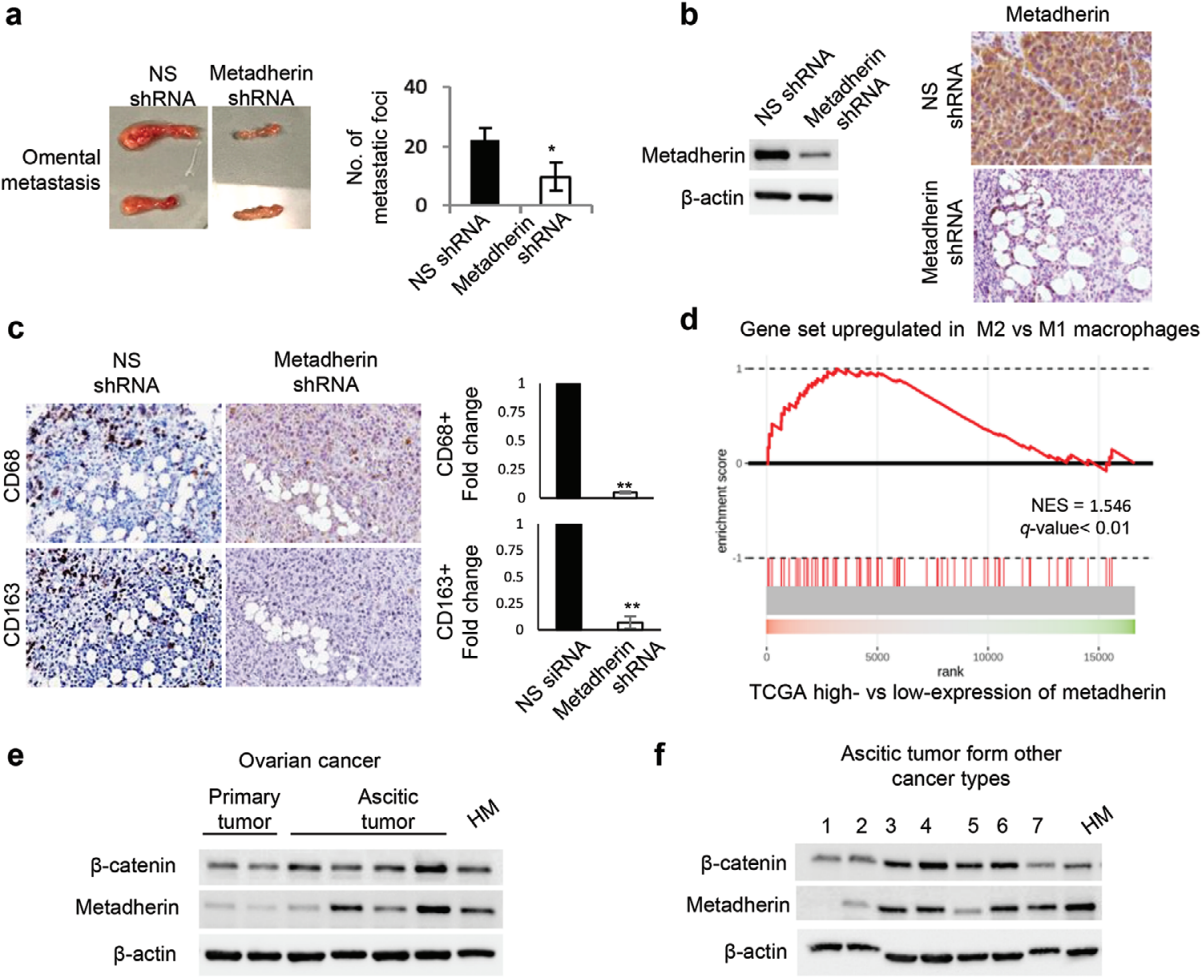
Knockdown of metadherin reduced tumor metastasis in humanized mice and its associations with *β*‐catenin and macrophages are observed in clinical samples. a) HM cells stably transfected with nonspecific (NS) or metadherin shRNA were orthotopically implanted into the ovarian bursa of humanized mice (3 mice per group). Omental metastasis and the number of mesenteric metastatic foci were analyzed. b) Successful knockdown of metadherin was confirmed with Western blot (left panel) and immunohistochemistry (right panel). *β*‐actin serves as a loading control. c) Immunohistochemistry was performed on consecutive tumor sections to stain for CD68+CD163+ tumor‐associated macrophages in the omentum, and their numbers were quantified and compared. d) Gene Set Enrichment Analysis was performed to compare the differentially upregulated genes in M2 versus M1 macrophages with the ranked gene list of metadherin‐high versus ‐low samples from TCGA. e) Co‐expression of *β*‐catenin and metadherin is observed in both ovarian cancer patient primary (lane 1 and 2: high grade serous) and ascitic samples (lane 3,5: mucinous; lane 4, 6: endometroid). f) Coexpression of *β*‐catenin and metadherin is observed in ascitic samples originated from other primary tumor types (lane 1, 5, 7: Uterus; 2, 3, 4: Stomach; 6: Colon; 8: Lung). *, *P* < 0.05, **, *P* < 0.01.

**Figure 6:**
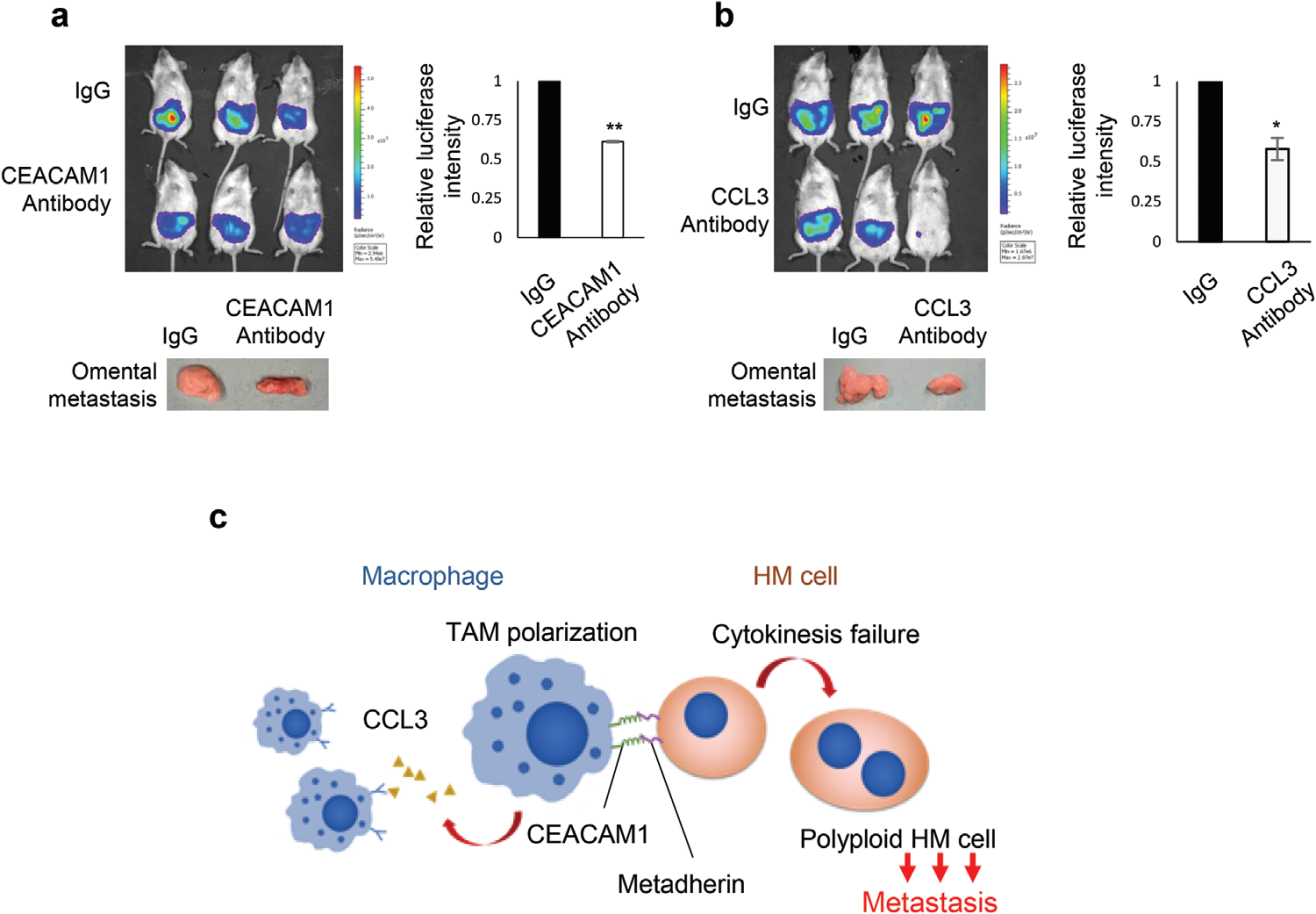
Neutralization of CEACAM1 or CCL3 in humanized mice reduced metastasis. HM cells were i.p. injected into humanized mice. One week after tumor inoculation, mice were treated with a) anti‐human CEACAM1 or b) anti‐human CCL3 antibody (200 mg/kg/mouse) every alternate day for five times. Corresponding isotype controls were used (3 mice per group). Peritoneal metastasis was visualized by bioluminescence imaging of luciferase activity. Mice were sacrificed 2 d after the final treatment and omental metastasis was shown. c) Schematic diagram showing the proposed interaction between HM and macrophages.

To test the role of metadherin, HM cells expressing nonspecific or metadherin shRNA were orthotopically injected into the ovarian bursa of humanized mice. Metadherin knockdown significantly decreases omental metastasis and the number of tumor nodules in these mice (2.3‐fold) (**Figure** **5**a). Western blot and immunohistochemical analysis of tumor samples indicated successful knockdown of metadherin and reduction of metadherin by *β*‐catenin knockdown (Figure 5b). Moreover, fewer CD68^+^CD163^+^ infiltrating TAMs were found in tumors with metadherin knockdown (Figure 5c). Gene Set Enrichment Analysis of differentially upregulated genes in M2 versus M1 macrophages against the ranked gene list of TCGA ovarian cancer samples with high versus low expression of metadherin also showed enrichment of M2‐like macrophage signature in the metadherin‐high samples (Figure 5d). We also observed coexpression of *β*‐catenin and metadherin in both primary and ascitic tumor samples from ovarian cancer patients by Western blot (Figure 5e). Interestingly, coexpression of *β*‐catenin and metadherin could also be detected in malignant ascites originating from other primary cancer types such as gastric and endometrioid cancers (Figure 5f). Furthermore, higher expression of *β*‐catenin and metadherin was associated with the high‐risk patient group with poorer overall survival (Figure S5, Supporting Information). These data together confirmed the importance of *β*‐catenin/metadherin in HM in recruiting TAMs at the pathological level. To validate the roles of CCL3 and CEACAM1 in humanized mice, we treated the mice with neutralizing antibodies after orthotopic injection of HM cells. Neutralization of either CCL3 or CEACAM1 reduced peritoneal metastasis in both humanized and nude mice (**Figure** **6**a,b; Figure S6, Supporting Information), hence implying the therapeutic potential of these molecules. Our proposed working model of HM–macrophage interaction that promotes metastasis is summarized in Figure 6c.

## 3 Discussion

Phenotypic heterogeneity is critical in determining the dynamics of tumor progression. Identifying the driver cell subsets that fuel heterogeneity and tumor metastasis will be crucial for developing effective treatments. Here we show for the first time that metastasis‐competent cells overexpressing *β*‐catenin can survive and progress selectively in a macrophage‐rich microenvironment at the metastatic site via juxtacrine activation of the metadherin/CEACAM1‐CCL3 signaling pathway and a significant subset of these cells are capable of generating polyploidy. Despite polyploidy being a hallmark of cancers that restricts the effectiveness of therapies, whether and how the tumor microenvironment causes these cells to form are poorly understood. Furthermore, while immunosurveillance by T cells to eliminate polyploid cancer cells has been reported,^[^ ^36^ ^]^ we are the first to identify the de novo induction of polyploid cancer cells by macrophages. Inability of the immune system to clear this polyploid subset would therefore contribute to disease progression.

Polyploidy provides an adaptive potential and survival advantage in a wide variety of processes.^[^ ^37^ ^]^ In cancers, polyploidy can result in chromosome instability that increases the evolution in tumor genome, tolerance to chromosome segregation errors, and resistance to drug treatments.^[^ ^38^ ^]^ In fact, nearly 30% of human cancers present genome‐wide duplication events and polyploid tumors are associated with poor prognosis.^[^ ^39^, ^40^ ^]^ These therapy‐resistant polyploid cancer cells represent exploitable targets to suppress tumor evolution, yet therapeutic targeting of this subpopulation is still underdeveloped. High‐throughput screening has led to the identification of resveratrol and aspirin as antitetraploid agents, which selectively kill tetraploid cells via AMPK activation;^[^ ^41^ ^]^ while a two‐step screen has revealed DPBQ as a novel lead compound that selectively activates p53‐dependent apoptosis in the high‐ploidy breast cancer cells.^[^ ^40^ ^]^ Other research groups took advantage of the increased metabolic demand of the polyploid cells.^[^ ^42^, ^43^ ^]^ While these studies have provided an important basis for translating the polyploid‐selective treatment into the clinic, an effective treatment regimen will also depend on the ability to define the driving forces for tumor cell karyotype diversity.^[^ ^44^ ^]^ It would be of great importance to delineate if there could be a dysregulated polyploid checkpoint in the highly metastatic cells and how they tolerate the presence of extra chromosomes since such data will further shed light on specific targeting of the polyploid population.

Juxtacrine signaling occurs through direct cell–cell contact, whereas paracrine signaling is conducted through soluble factors. Integration of these signals would allow immediate responses of cells to its microenvironment. Although macrophages and cancer cells are known to be in close contact in vivo,^[^ ^9^ ^]^ much less is known about juxtacrine signaling between macrophages and cancer cells in contrast to paracrine signaling pathways conducted through chemokines. Only a few contact‐dependent pathways have been reported thus far, which include EphA4/Ephrin and CD90/CD11b signaling pathways for breast cancer stem cells_,_ and an ICAM1/integrin signaling for ovarian cancer spheroids.^[^ ^45^, ^46^ ^]^ Therefore, the metadherin/CEACAM1 signaling between metastatic cells and macrophages identified here has provided further new insights in targeting this tumor–immune interaction.

While metadherin (named for metastasis adhesion protein) was first reported to be a transmembrane protein containing a “lung homing domain” that mediates lung metastasis of mouse mammary tumor cells,^[^ ^23^ ^]^ it was later reported to reside in the cytosol, endoplasmic reticulum, nucleus and nucleolus, and its subcellular localization is closely linked to its various tumor‐promoting functions.^[^ ^24^, ^42^ ^]^ How the context‐dependent localization of metadherin is regulated remains largely unresolved. Here using a biotinylation assay and subsequent Western blotting, we demonstrated the upregulation of the relatively uncommon cell surface metadherin in the highly metastatic ovarian cancer cells. High expression of metadherin has been observed in numerous cancer types.^[^ ^47^ ^]^ In ovarian cancer, it significantly correlates with enhanced peritoneal and lymph node metastases, chemoresistance, and also poor survival.^[^ ^48^, ^49^ ^]^ Moreover, mice with genetic metadherin ablation are viable and fertile, suggesting that metadherin could be highly tumor specific.^[^ ^50^ ^]^

We have also identified CEACAM1 as an important modulator of macrophage and HM interactions. CEACAM1 is expressed on the surface of different myeloid cells and lymphocytes and is regarded as a promising target for immunotherapy.^[^ ^27^, ^51^ ^]^ CM‐24, a monoclonal antibody targeting CEACAM1, has been evaluated clinically for treating advanced or recurrent cancers,^[^ ^52^ ^]^ and is currently under early clinical trial for combination with Nivolumab, an anti‐PD1 antibody (NCT04731467). In fact, we also found that CEACAM1 may regulate PD‐L1 expression in macrophages (data not shown), which further implicates the clinical potential for targeting CEACAM1. The chemokine CCL3 is emerging as a potent activator of both innate and adaptive responses. For example, CCL3 plays a central role in the recruitment of distinct immune cells to the tumor site, dendritic cell homing, and antigen‐specific T‐cell immunity.^[^ ^34^ ^]^  In addition to targeting CEACAM1 and CCL3, altering the polarization state of macrophages by nanoparticles would also represent a promising direction for macrophage‐based immunotherapy given our finding that metastatic cells could induce TAM‐like phenotypes.^[^ ^53^, ^54^ ^]^

In summary, targeting the highly evolvable, therapy‐resistant polyploid tumor cell subsets likely has great therapeutic potential. Several studies, including ours, have suggested that Wnt/*β*‐catenin signaling is higher in ovarian cancer metastatic sites than in the primary sites using different approaches.^[^ ^55^, ^56^, ^57^ ^]^ A detailed analysis of this signaling pathway is therefore warranted. Furthermore, although *β*‐catenin is at present undergoing clinical trials as an anticancer target, serious side effects are often observed as it is an essential component in normal physiologic homeostasis. Our findings may have implications for the use of its downstream targets as therapeutics. These findings are not only relevant to ovarian cancer, but also to other tumor types where *β*‐catenin/metadherin overexpression and macrophage cooption are important for the pathological process.

## 4 Experimental Section

### Cell Lines and Cell Culture

The human ovarian cancer cell line HEYA8 was a gift from Dr J. Liu (MD Anderson Cancer Centre, Houston, TX). The NM and HM sublines were derived and transduced with firefly luciferase as previously described and maintained in RPMI medium (Invitrogen, Carlsbad, CA).^[^ ^13^ ^]^ The monocytic leukemia cell line THP1 was also kept in RPMI1640 medium. Media were supplemented with 100 units mL^‐1^ penicillin, 100 mg mL^‐1^ streptomycin and 5% fetal bovine serum (FBS). All cultures were maintained in a humidified incubator at 37 °C with 5% CO_2_. For THP1 differentiation, cells were treated with 100 × 10^‐6^ m phorbol 12‐myristate 13‐acetate (PMA) for 48 h. Cell was then polarized to classical macrophages with lipopolysaccharides (LPS; Calbiochem, San Diego, CA; 10 ng mL^‐1^) and interferon‐*γ* (IFN‐*γ*; R&D Systems, Minneapolis, MN; 20 × 10^‐9^ m) or to alternative macrophages with IL4 (25 µg mL^‐1^) for another 24 h. PMA and polarizing agents were removed and cultured in a complete medium for 24 h before coculture.

### Small Interfering RNA (siRNA)‐Mediated Knockdown

Nonspecific, *β*‐catenin, metadherin, and p65 siRNAs were purchased from Dharmacon (Lafayette, CO). Cells were transfected with 20 nmol L^‐1^ siRNA using siLectFect (Bio‐Rad, Hercules, CA) as instructed by the manufacturer.

### Generation of Stable Cell Line

HEK293T cells were cotransfected with GFP‐tagged histone H2B and packaging vectors using the X‐tremeGENE transfection reagent (Roche Applied Science, Upper Bavaria, Germany). Conditioned media containing the recombinant viruses were collected and filtered through 0.45 µm pore‐size filters. The filtrate was then used for transduction in the presence of 4 µg mL^‐1^ polybrene (Sigma, St. Louis, MO, USA). Cells were selected with 1 µg mL^‐1^ puromycin. Metadherin short hairpin (sh) RNA was obtained from Sigma and *β*‐catenin shRNA was previously reported.^[^ ^13^ ^]^

### RNA Sequencing and TCGA Data Analysis

Total RNA was extracted from HM and NM cells with Trizol Reagent (Invitrogen). RNA sequencing was performed at the Centre for PanorOmic Sciences (CPOS, The University of Hong Kong). The top 100 upregulated protein‐coding genes (with lowest FDR and highest fold‐change) in HM versus NM were used for constructing gene regulatory networks based on gene–gene interactions in the TCGA ovarian serous cystadenocarcinoma dataset inferred by ARACNE. For functional enrichment of KEGG pathways, the overrepresentation analysis (ORA) was used to evaluate the statistical significance of the overlap between the network gene list and the genes in the gene sets based on the hypergeometric test. To access the correlation with Wnt signaling in patients, samples were first ranked by the 100‐gene signature and then compared to the z‐score for Wnt‐*β*‐catenin signaling hallmark. To test for association of metadherin and macrophages in patient samples, Gene Set Enrichment Analysis of differentially upregulated genes in M2 versus M1 macrophages was compared with the ranked gene list with high versus low expression of metadherin in TCGA samples.

### Western Blot Analysis

To separate the two cell types from the coculture, THP1 macrophages were positively selected using CD14 microbeads (Miltenyi Biotec, Bergisch Gladbach, Germany). Cells were lysed with NP‐40 lysis buffer (50 × 10^‐3^ m Tris HCl (pH 8.5), 150 × 10^‐3^ m NaCl and 1% NP‐40). Total proteins were separated with 7.5% or 10% polyacrylamide gels and transferred onto nitrocellulose membranes. The membrane was then blocked with non‐fat dry milk for an hour and incubated with antibodies at 4 °C for 1 h to overnight. The membrane was washed thrice and incubated with appropriate secondary antibodies conjugated with peroxidase. Proteins bands were detected by Western‐Lightning Plus Enhanced Chemiluminescence (Perkin Elmer), and their intensities were determined by densitometry using the ImageJ software. Primary antibodies were obtained as follows: *β*‐catenin (BD Transduction Laboratories, San Diego, CA; 1: 5000), metadherin (Abcam, Cambridge, UK; 1:5000), CEACAM1 (Cell Signaling Technology, 1:1000), *β*1 integrin (clone JB1A) (Millipore; 1:1000), *β*‐actin (Sigma, 1:5000). HRP‐conjugated secondary antibodies were purchased from Bio‐rad (1:3000).

### Total RNA Extraction, Reverse Transcription (RT) and Semiquantitative RT‐PCR

RNA was extracted with Trizol Reagent (Invitrogen) and reverse‐transcribed into cDNAs using the M‐MLV Reverse Transcriptase (Invitrogen). Specific sequences of primers are as follows: *β*‐catenin (Forward 5′‐GGCTACGTCCAGGAGCGCA‐3′; Reverse: 5′‐ GCTGCACAAACAATGGAATGG‐3′), metadherin (Forward: 5’‐ GGCAATTGGGTAGACGAAGA‐3’; Reverse: 5’‐ CCTGTTTTGGACGGGTTTTA‐3’), CCL3 (Forward: 5’‐ATTCCGTCACCTGCTCAGAA‐3’; Reverse: 5’‐TAGGAAGATGACACCGGGCTT‐3’), CCL18 (Forward: 5’‐ TCTGCTGCCTCGTCTATACCT‐3’; Reverse: 5’‐ AGCTTCAGGTCGCTGATGTA‐3’), CCL22 (Forward: 5’‐ ACAGAGCATGGATCGCCTA‐3’; Reverse: 5’‐ AAGGTTAGCAACACCACGCCA‐3’), IL‐6 (Forward: 5’‐TGGCAG AAAACAACCTGAACC‐3’; Reverse: 5’‐TGGGTCAGGGGTGGTTATTG‐3’) and IL‐1*β* (Forward: 5’ CCACCTCCAGGGACAGGATA‐3’; Reverse: 5’‐TGGGATCTACACTCTCCAGC‐3’) and CD163 (Forward: 5’‐ GCGGCTTGCAGTTTCCTCAA‐3’; Reverse: 5’‐ CTCAGAATGGCCTCCT TTTCCA‐3’) and GAPDH (Forward: 5’‐ATGTTCGTCATGGGTGTGAACCA‐3’; 5’‐TGGCAGGTTTTTCTAGACGGCAG‐3’).

### Migration Assay

Fifty thousand cells, resuspended in serum‐free medium, were added into transwell inserts (Millipore). Conditioned medium was added to the lower chambers. After 4 h, cells were fixed with ice‐cold methanol and were stained with 0.5% crystal violet. Cells that have not penetrated through the membrane pores were removed and the number of migrated THP1 cells was then determined using MTT assay. Briefly, MTT (3‐(4,5‐dimethylthiazol‐2‐yl)‐2,5‐diphenyltetrazolium bromide) (Sigma, St. Louis, MO) solution (5 mg mL^‐1^) was added to each well and plates were incubated for 2 h for the reduction of MTT to formazan by viable cells. The insoluble formazan was dissolved with DMSO and the color intensities were monitored with a microplate reader at 570 nm (Bio‐Rad).

### Sphere Forming Assay

Tumor sphere formation assays were conducted as previously described with slight modifications.^[^ ^13^ ^]^ Briefly, 2500 cells mL^‐1^ were plated in ultralow attachment 100 mm culture plate in serum‐free stem cell‐selective medium. After 3 d, each plate was examined and photographed using light microscope and the number of spheres was counted.

### Immunofluorescence

Glass coverslips were placed in each well of a 12‐well plate before cell plating. At the time of harvest, cells were washed twice with PBS and then fixed with 4% paraformaldehyde. Permeabilization was done with 0.1% Triton for 5 min, followed by 15 min blocking with 5% bovine serum albumin (BSA). The coverslips were incubated with primary antibodies for 1 h, followed by three washes with PBS and incubation with appropriate secondary antibodies for another hour. After washing with PBS thrice, the coverslips were mounted on glass slides with Vectashield mounting medium containing DAPI as a nuclear counterstain (Vector Laboratories, Burlingame, CA). For surface protein staining, the fixation and permeabilizing steps were done after incubation with antibodies, and the whole experiment was carried out on ice.  Primary antibodies used are as follows: metadherin (Abcam), *β*‐catenin (BD Transduction Laboratories), N‐cadherin (Sigma), *α*‐tubulin (Santa Cruz Biotechnologies, Dallas, TX, USA) and F4/80 (Cell Signaling Technology).

### Senescence *β*‐Galactosidase Staining

Senescence *β*‐galactosidase staining was performed using the senescence *β*‐galactosidase staining kit according to the manufacturer's protocol (Cell Signaling Technology). Briefly, cells were fixed with a fixative solution for 15 min at room temperature, followed by PBS wash and incubation with *β*‐galactosidase staining solution at 37 °C at least overnight in a dry incubator. The development of blue color was observed under light microscope.

### EdU Staining

EdU staining was performed with the Click‐iT EdU Cell Proliferation kit (Alexa Fluor 594 dye) according to the manufacturer's instructions (Invitrogen). Briefly, cells were incubated with the EdU, which could be incorporated the newly synthesized DNA during cell growth. After incubation, cells were fixed and permeabilized, followed by EdU detection with the Click‐iT EdU staining cocktail containing the Alexa Fluor azide. Nuclei were counterstained with Hoechst 33342.

### Time‐Lapse Microscopy

Cells were cultured in CO_2_‐independent medium (Invitrogen), supplemented with 10% FBS, 100 units mL^‐1^ penicillin, and 100 µL streptomycin, and 4 × 10^‐3^ m L‐glutamine. Cells were observed with an inverted microscope using a 10× objective (numerical aperture, 0.95; Nikon) in a chamber maintained at 37 °C. Each well was imaged every 10 min using a motorized stage. Time‐lapse images were viewed using MetaMorph or ImageJ and the phenotypes were scored and analyzed.

### Cytokine Array

Conditioned media were analyzed with Human Cytokine Array Kit (Panel A; R&D Systems) according to the manufacturer's instructions. The array detects the relative levels of 36 different cytokines and chemokines. Briefly, cytokine array membranes blocked with blocking buffer for 1 h were incubated with sample/antibody mixture overnight at 4 °C on a rocking platform shaker. The membranes were washed thrice with wash buffer, followed by incubation with diluted streptavidin–HRP for 30 min. After three more washes, proteins on the membranes were detected by chemiluminescence.

### Enzyme‐Linked Immunosorbent Assay (ELISA)

Concentration of CCL3 in the culture supernatant was measured with the SimpleStep CCL3 ELISA kit according to the manufacturer's protocol (Abcam). Briefly, diluted samples were incubated with the CCL3 capture and detector antibody cocktail in a precoated microplate. After 1 h, the wells are washed three times, followed by incubation with TMB development solution for 10 min. Stop solution was finally added and OD at 450 nm was measured.

### In Vivo Studies

All mouse studies were performed in accordance with protocols approved by Committee on the Use of Live Animals in Teaching and Research (The University of Hong Kong) and the Animals (Control of Experiments) Ordinance of Hong Kong (CULATR‐5297‐20). Female athymic nude mice were purchased from the Charles River Laboratories (Wilmington, MA, USA) and humanized NSG mice were purchased from the Jackson Laboratory (Bar Harbor, ME, USA). All mice were maintained at the Centre for Comparative Medicine Research (The University of Hong Kong). For intraperitoneal (i.p.) injection, 10^6^ cancer cells were injected into mice. For orthotopic injection, 10^5^ cancer cells were injected into the ovarian bursa of mice. To monitor cancer progression in vivo, mice were injected with 200 µL of 30 mg mL^‐1^ D‐luciferin (Perkin Elmer, Waltham, MA), and images were acquired starting 10 min after luciferin injection using a Xenogen IVIS 100 cooled CCD camera (Xenogen, Alameda, CA, USA). For macrophage depletion, one week after i.p. tumor inoculation, mice were i.p. injected with clodronate liposomes (100 µl per mouse; Liposoma, Amsterdam, the Netherlands) twice a week for two weeks. PBS liposomes were used for control mice. For CEACAM1 and CCL3 neutralization, one week after tumor inoculation, huNSG mice were i.p. injected with human CEACAM1 antibody (20 mg kg^‐1^; R&D Systems) or human CCL3 antibody (20 mg kg^‐1^; Biolegend, San Diego, CA, USA) every alternate day for five times. Tumor‐bearing nude mice were similarly treated with mouse CEACAM1 antibody (20 mg kg^‐1^; Biolegend) or mouse CCL3 antibody (20 mg kg^‐1^; R&D Systems). Corresponding isotype controls were used. Mice were sacrificed 2 d after the final treatment. At the time of sacrifice, the number of all visible (>0.1 cm) metastatic nodules in the peritoneal cavity was counted. Tissue samples were harvested, fixed with 10% formalin and embedded in paraffin for histological analysis. For in vivo live cell tracking, HM cells were stained with CellTracker Red CMTPX fluorescent dye (Invitrogen) according to the manufacturer's instructions, followed by i.p. injection into nude mice. After 4 h, the mice were sacrificed and the omentum was extracted and fixed with 4% paraformaldehyde for immunostaining.

### Human Ovarian Cancer Samples

Formalin‐fixed and paraffin‐embedded clinical samples of paired ovarian high‐grade serous carcinoma and its metastasis were obtained from the pathology archives of Department of Pathology, The University of Hong Kong, Queen Mary Hospital, for immunohistochemical analyses. Clinicopathological information of patients was provided in Table S1 (Supporting Information). Fresh primary (2 ovarian cancer) and ascitic samples (4 ovarian, 3 endometrioid, 3 gastric, 1 colon, and 1 lung cancer samples) (at least 70% tumor cells) were also obtained from Queen Mary Hospital for Western blot analyses. The use of these specimens was approved by the Institutional Ethical Review Board for Research on the use of human subjects at the University of Hong Kong (EA1506047).

### Immunohistochemistry

Paraffin sections from patient or mice tumors were deparaffinized with xylene and rehydrated in graded ethanol. After blocking and heat‐induced antigen retrieval using citrate buffer, the specimens were separately incubated with primary antibodies against *β*‐catenin, metadherin, CD68 (human macrophages), CD163 (human M2 marker), F4/80 (mouse macrophages), or rabbit IgG control (Cell Signaling) overnight at 4°C.^[^ ^58^ ^]^ The slides were further processed using rabbit specific HRP/DAB (ABC) detection IHC kit according to the manufacturer's protocol (Abcam). Quantification was performed using ImageJ Fiji^[^ ^59^ ^]^.

### Statistical Analysis

All experiments were repeated at least twice in duplicate. Each of them yields essentially similar results. The significance of the data was analyzed by paired Student's T‐test (GraphPad Software). *P* < 0.05 was considered significant.

## Conflict of Interest

The authors declare no conflict of interest.

## Author Contributions

S.K.Y.T. conducted the experiments, analyzed results, and prepared the manuscript. M.K.S.T. assisted the mice experiments. Y.T. and J.Z. performed bioinformatic analysis. K.L.L.C. and P.P.C.I. provided the patient samples. J.S. and A.S.T.W. designed and supervised the project.

## Supporting information

Supporting Information

Supplemental Video 1

## Data Availability

The data that support the findings of this study are available in the supplementary material of this article.
