# Supplementary material for: A Selective β−Catenin‐Metadherin/CEACAM1‐CCL3 Axis Mediates Metastatic Heterogeneity upon Tumor–Macrophage Interaction
Source: Adv Sci (Weinh). 2022 Apr 11;9(16):2103230. doi: 10.1002/advs.202103230 (PMC9165500; doi:10.1002/advs.202103230)
Supplement: Supplementary file 1 — Supporting Information [file ADVS-9-2103230-s002.pdf]

ADVANCED SCIENCE
Open Access

Supporting Information

for *Adv. Sci.*, DOI 10.1002/advs.202103230

A Selective β–Catenin-Metadherin/CEACAM1-CCL3 Axis Mediates Metastatic Heterogeneity upon Tumor–Macrophage Interaction

Sally K. Y. To, Maggie K. S. Tang, Yin Tong, Jiangwen Zhang, Karen K. L. Chan, Philip P. C. Ip, Jue Shi and Alice S. T. Wong**

Characteristics	Number
High grade serous ovarian carcinoma	10
FIGO stage	
IIIB	1
IIIC	5
IV	4
Age (range 35-78)	
<60	7
≥60	3
Site of metastatic biopsy	
Omentum	9
Bladder flap	1

Supplementary Table 1. Clinical characteristics of paired primary and metastatic samples from patients.

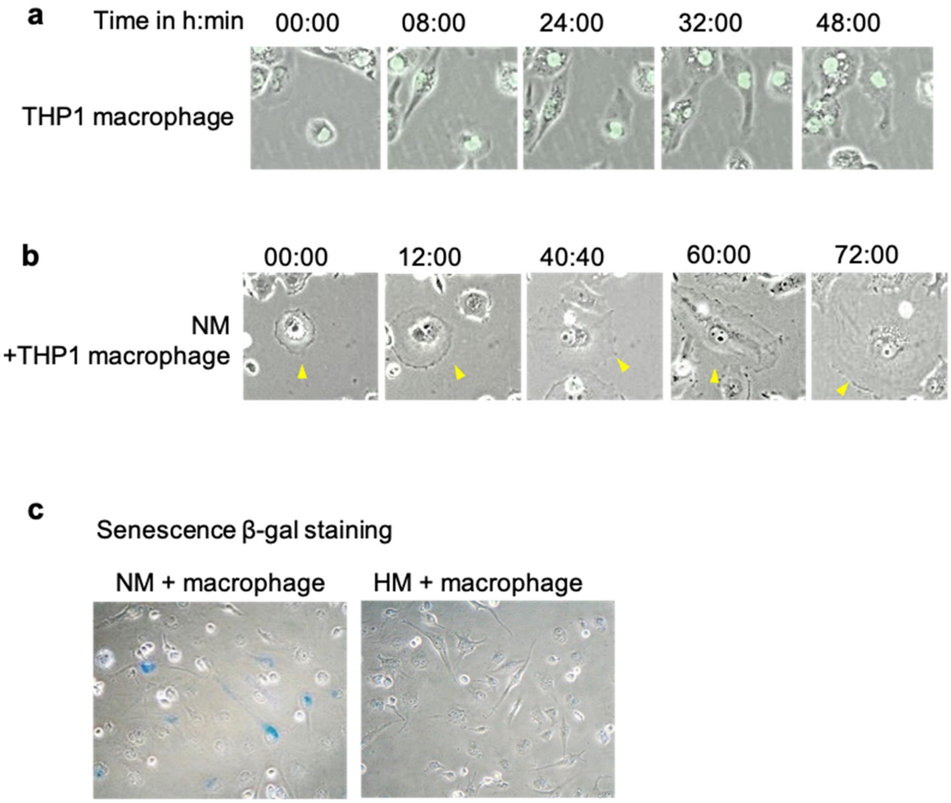

Figure S1. NM entered senescence when cocultured with macrophages. a, Phototoxicity was not observed in THP1 macrophages alone during the time-lapse imaging. **b**, Representative time-lapse images of growth arrested NM cocultured with macrophages for 72 hours were shown. **c**, NM cocultured with macrophages were positive for senescence β-galactosidase (SA-β-gal) staining.

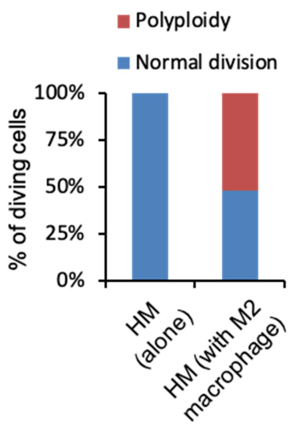

Figure S2. M2 macrophages promotes HM polyploidy. THP1 macrophages were polarized to M2 phenotype by IL4, followed by coculture and time-lapse imaging. At least 30 cells were traced for each group, and the percentages of HM cells undergoing normal division or polyploidization were shown.

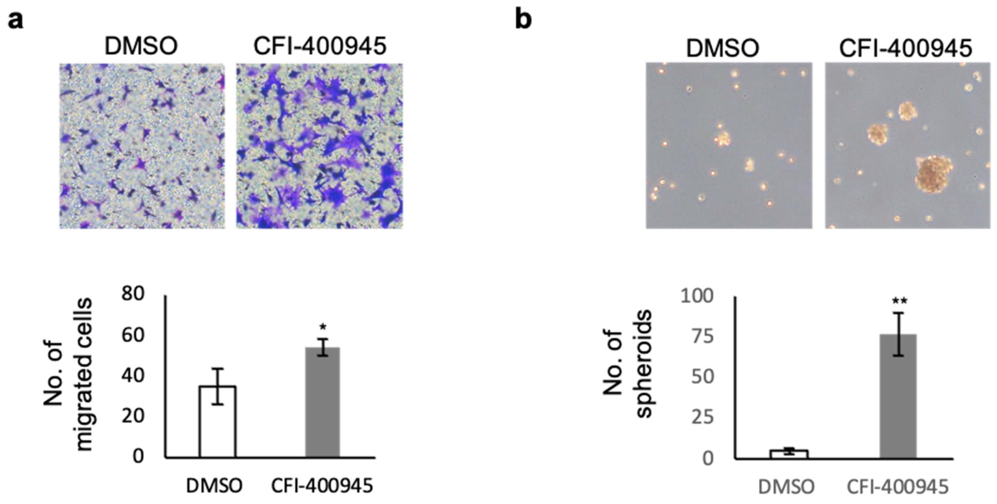

Figure S3. Chemically-induced polyploid HM cells are functionally more aggressive. HM cells were pre-treated CFI-400945 (100nM), a PLK4 inhibitor, for 48 hours to induce polyploidy. DMSO was used as a vehicle control. Treated cells were then used for **a,** migration assays, or **b,** sphere formation assays. *, P <0.05, **, P< 0.01.

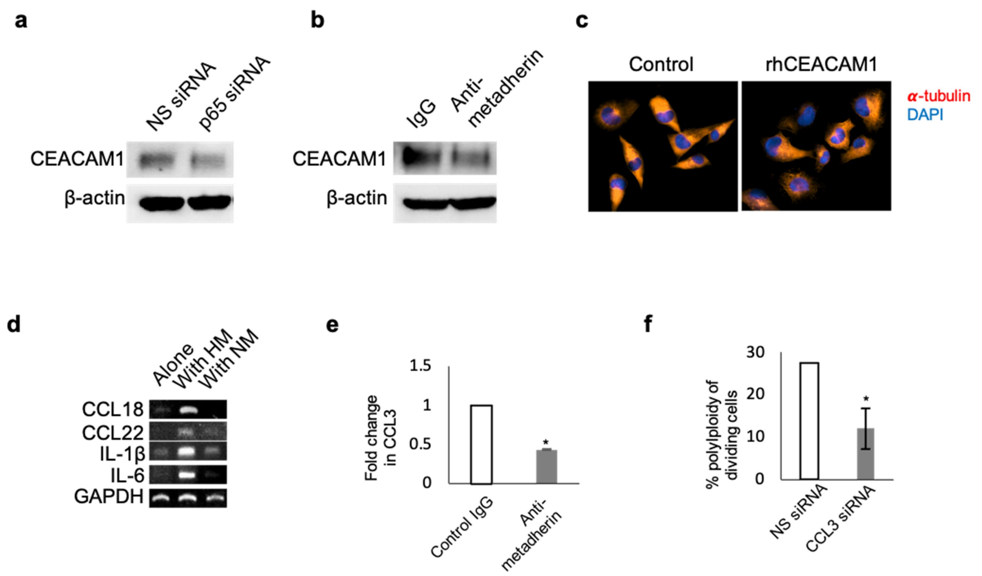

Figure S4. Characterization of the CEACAM1-Metadherin/CCL3 signaling. a, THP1 macrophages were transfected with nonspecific (NS) or p65 siRNA and CEACAM1 expression levels were analyzed by Western blot. β-actin served as a loading control. **b,** THP1 macrophages were cocultured with HM cells pretreated with IgG or metadherin antibody (R&D systems). CEACAM1 expression levels in the isolated macrophages were analyzed. **c**, HM cells were treated with recombinant human (rh) CEACAM1. Cells were fixed and stained with α-tubulin (red), with DAPI visualizing the nuclei. **d,** After coculture with HM for 48 hours, RNA was extracted from the isolated macrophages and RT-PCR was performed to determine the expression levels of CCL18, CCL22, IL-1β and IL6. GAPDH serves as a loading control. **e**, CCL3 levels in the coculture supernatants were analyzed by ELISA. **f,** THP1 macrophages were transfected with NS or CCL3 siRNA, followed by HM coculture and time-lapse imaging. At least 30 cells were traced for each group, and the percentages of HM cells undergoing normal division or polyploidization were shown. *, *P* <0.05.

a

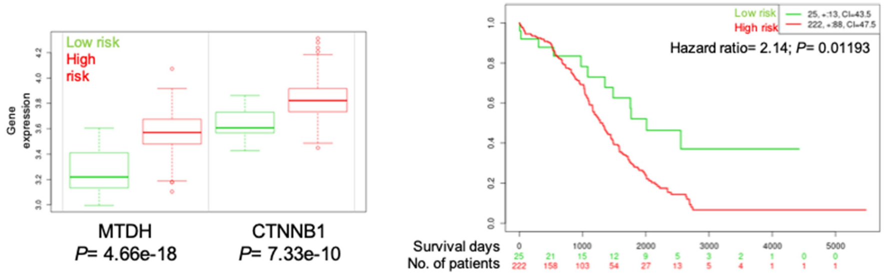

b

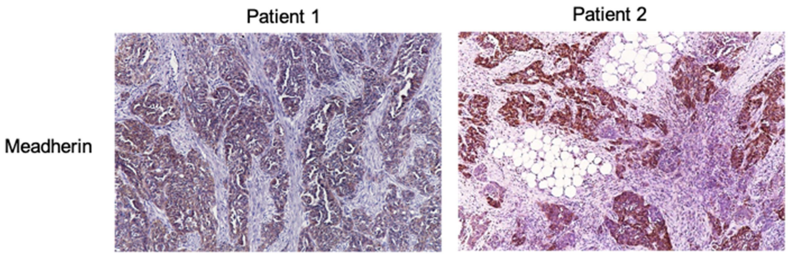

Figure S5. Clinical relevance of β-catenin, metadherin in patients. a, Higher expression of β-catenin and metadherin is associated with patients in the high-risk patient group of ovarian cancer patients with poorer overall survival. Results were obtained from SurvExpress (http://bioinformatica.mty.itesm.mx/SurvExpress; TCGA-ovarian serous cystadenocarcinoma June 2016, n=247). **b,** Immunohistochemistry shows high metadherin expression by cancer cells in metastatic samples from two ovarian cancer patients.

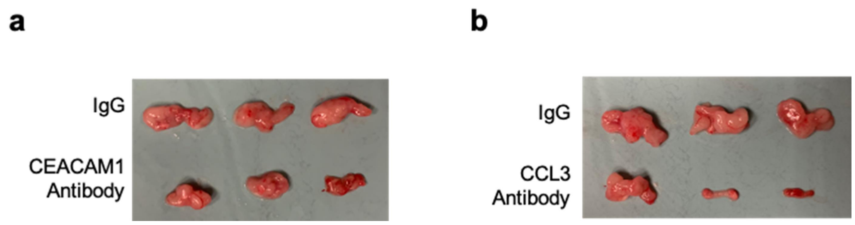

Figure S6. Neutralization of CCL3 or CEACAM1 in nude mice reduced metastasis. HM cells were i.p. injected into humanized mice. One week after tumor inoculation, mice were treated with **a,** anti-mouse CEACAM1 or **b,** anti-mouse CCL3 antibody (200 mg/kg/mouse) every alternate day for 5 times. Corresponding isotype controls were used (3 mice per group). Mice were sacrificed two days after the final treatment and omental metastasis were shown.

Video S1. The white arrow indicates an HM cell undergoing normal division, whereas the yellow arrow indicates an HM cell becoming polyploid. THP1 macrophages express histone H2B-GFP (green) (time in h:m:s).
